# A sericin/ graphene oxide composite scaffold as a biomimetic extracellular matrix for structural and functional repair of calvarial bone

**DOI:** 10.7150/thno.39502

**Published:** 2020-01-01

**Authors:** Chao Qi, Yan Deng, Luming Xu, Cheng Yang, Yuanyuan Zhu, Guobin Wang, Zheng Wang, Lin Wang

**Affiliations:** 1 Department of Clinical Laboratory, Union Hospital, Tongji Medical College, Huazhong University of Science and Technology, Wuhan, 430022, China.; 2 Research Center for Tissue Engineering and Regenerative Medicine, Union Hospital, Tongji Medical College, Huazhong University of Science and Technology, Wuhan, 430022, China.; 3 Department of Gastrointestinal Surgery, Union Hospital, Tongji Medical College, Huazhong University of Science and Technology, Wuhan, 430022, China.

**Keywords:** Sericin, Graphene oxide, Bone regeneration, Bone marrow-derived mesenchymal stem cells, Migration and osteogenesis differentiation

## Abstract

Bone defects affect millions of people worldwide each year, leading to severe disabilities. Biomimetic scaffolds mediated tissue regeneration represents a promising alternative for bone repair. However, the major problem associated with most currently clinical available artificial bone substitutes (scaffolds) is that they mainly possess filling function but lack of osteo-induction abilities. Therefore, development of biomaterials with osteo-induction property for effective bone regeneration is highly desired.

**Methods:** We report the design and fabrication of a photo-crosslinked sericin methacryloyl (SerMA)/ graphene oxide (GO) hydrogel (SMH/GO) as a biomimetic scaffold for the functional repair of the bone. The mechanical strength, degradation and biocompatibility behavior of SMH/GO hydrogel were measured *in vitro*. The effect of SMH/GO hydrogel on BMSCs proliferation, migration, osteogenesis differentiation was assessed. After that, SMH/GO-2 was used as an artificial bone substitute for bone regeneration after calvarial defects and effect on bone repair was evaluated by histological, X-Ray and microCT analysis. Furthermore, the potential mechanism of SMH/GO hydrogel regulating BMSCs migration and differentiation was investigated by RNA sequencing.

**Results:** This scaffold has good biocompatibility, cell adhesive property, proliferation- and migration-promoting effects, and osteogenic induction property. After being implanted in a rat calvarial defect model, this SMH/GO scaffold effectively promotes new bone regeneration and achieves structural and functional repair within 12 weeks by inducing autologous bone marrow-derived mesenchymal stem cells (BMSCs) differentiation. By utilizing cell-biological assays and RNA sequencing, we reveal its possible regeneration mechanisms: the SMH/GO hydrogel regulates BMSCs migration and osteo-differentiation via activating MAPK, TNF, and chemokine signaling for bone regeneration.

**Conclusion:** Aiming to meet clinical demands and overcome current limitations of existing artificial bones, we have developed a new type of sericin/ graphene oxide composite scaffold and provided histological, functional, and molecular evidence demonstrating that it is capable of effectively repairing defective bones by inducing autologous BMSCs directional migration and osteogenic differentiation.

## 1. Introduction

Bone, one of the most abundant tissues in human body, mainly serves to provide mechanical support, protect organs, produce hematopoietic cells, and deposit minerals 1. It is easily damaged by trauma, osteoporosis, arthritis, tumor or other diseases 2. Approximately 2.2 million bone grafting surgeries are performed annually worldwide, resulting in astronomical health care expenses (over $ 2.5 billion per year in the United State) 3. Although bone has certain self-repairing and regenerative capacity, clinical treatments are usually required for severe bone injuries 4 because it may lead to infection, disability, or even death if left untreated.

Current gold standards for treating bone defects are autograft and allograft 5, which are limited by donor shortage or immunogenicity 6,7. Thus, tissue-engineered bone substitutes have become promising alternatives 8-12. Most of scaffolds are capable of filling bone defects but lack osteo-induction ability, which partially explains frequently-observed clinically unsatisfying bone repair. Bioactive scaffolds encapsulating exogenous cells 13 or growth factors 14 provide an osteogenic microenvironment for bone tissue regeneration 15, which however may cause immune rejection that could lead to high treatment costs 16. Although some calcium phosphate (CaP) bioceramics as a potential bone-replacing material possess certain osteo-induction ability 17, they may also induce immune rejections and their osteo-induction mechanisms remain unclear 18.

Seed cells are one of the key elements for constructing artificial bone substitutes 19. Bone mesenchymal stem cells (BMSCs), the progenitors of osteoblasts involved in bone formation process, have the capability of osteogenic differentiation 20. After bone injury, BMSCs migrate to the injury site in response to endogenous chemokines and then differentiate into osteoblasts following stimulation of bone morphogens 21. BMSCs as an important source of seed cells for bone regeneration 22 are often encapsulated in some bioactive scaffolds 15. However, implantation of exogenous BMSCs alone is challenged by immune rejection and its bone repair efficiency is limited 23, 24. In this study, we proposed to design and fabricate a new type of biomaterial with functions of promoting osteogenic differentiation and BMSCs directional migration for the repair of bone defects.

Sericin, a natural protein extracted from silk cocoons, has unique advantages for tissue regeneration, including low immunogenicity 25, good biocompatibility 26, biodegradability, and capability of promoting cell adhesion and proliferation 27. In previous studies, we introduced methacrylogy groups to sericin and prepared a photo-crosslinkable sericin hydrogel (SMH) for cartilage and skin regeneration. In these studies, SMH was found to facilitate BMSCs migration *in vivo* 28.

While the mechanical properties of sericin hydrogels are suitable for soft tissue repair, it can hardly meet the needs for bone repair. To address this problem, we fabricated a new SMH/GO composite hydrogel by combining graphene oxide (GO) with sericin. GO can provide tunable mechanical strength and has osteogenic induction capability 29-31. By varying GO concentrations, we could conveniently adjust mechanical properties of SMH/GO composite hydrogels to meet the requirements for bone regeneration. In addition, this composite hydrogel inherits sericin's advantageous properties, including good biocompatibility, cell-adhesion and cell-proliferation-promoting effects. After being implanted in a rat calvarial defect model, SMH/GO hydrogels accelerated bone regeneration via promoting migration of autologous BMSCs towards the implantation sites and inducing osteogenesis even without exogenous stimulating factors. Using RNA sequencing and bioinformatics analysis, we revealed the main pathways mediating migration and osteogenic differentiation of delivered BMSCs: MAPK, TNF and chemokine signaling pathways 32, 33. Together, our study highlights the therapeutic efficacy of the SMH/GO composite hydrogel on bone regeneration and reveals the underlying molecular mechanisms.

## 2. Materials and Methods

### 2.1 Materials

Natural fibroin-deficient mutant silkworm cocoons (*140 Nd-s*) were supplied by the Seri cultural Research Institute, China Academy of Agriculture Science (Zhenjiang, Jiangsu, China). Sodium carbonate (Na_2_CO_3_), methacrylic anhydride (MA) and graphite powder were purchased from Sinopharm Chemical Regent Co., Ltd. (Shanghai, China). Cellulose dialysis membranes (MWCO: 3500 Da) were obtained from Spectrum Laboratory, Inc (Rancho Dominguez, CA). 2-hydroxy-1-(4-(hydroxyethoxy) phenyl)-2-methyl-1-propanone (Irgacure2959) was purchased from Sigma-Aldrich (USA). Osteobone was purchased from Yenssen Biotech (Jiangsu, China). Osteocalcin (OCN), Collagen I (COL I), CD44 (15675-1-AP) and CD73 (12231-1-AP) antibodies were purchased from proteintech (China). Runx 2 antibody was purchased from Boster Biological Technology (China).

### 2.2 Isolation of silk sericin

Silk sericin was isolated from natural fibroin-deficient mutant silkworm cocoons (*140Nd-s*) by high heat/alkaline degumming method as previously described 34. Briefly, 1 g cocoons were cut into pieces and boiled in 30 mL of 0.02 M Na_2_CO_3_ solution for 1 hour. The solution was centrifuged at 3500 rpm for 5 min to remove the insoluble residue and then dialyzed (MWCO: 3500 Da) against distill water for 3 days at room temperature. Sericin was then obtained by lyophilization.

### 2.3 Synthesis of SerMA

SerMA was synthesized as previously described 35. Briefly, 1 g sericin was dissolved in PBS (pH 8.5) at room temperature. 8 hours later, 1 g methacrylic anhydride (MA) was added into sericin solution. The mixture was stirred for 12 hours at room temperature. Then, the solution was dialyzed (MWCO: 3500 Da) against PBS and distilled water to remove methacrylic anhydride. After that, SerMA was obtained by lyophilization.

### 2.4 Preparation of graphene oxide

Graphene oxide was prepared as previously described 36. In brief, 2 g graphite was dissolved in 46 mL of concentrated H_2_SO_4_, and KMnO_4_ (6 g) was subsequently added into the solution with stirring. The reaction mixture was stirred at 40 ^o^C for 6-8 hours. Subsequently, 96 mL ddH_2_O was added and the solution was stirred for another 40 min. 300 mL ddH_2_O was added to terminate the reaction. After 5 mL 35% H_2_O_2_ was added, this mixture was suspended in 5% HCl solution and separated by centrifugation. Finally, graphene oxide was obtained after washing with ddH_2_O for three times.

### 2.5 Fabrication of SMH/GO composite hydrogel

8 mg graphene oxide (GO) was suspended in 2 mL distilled water, and sonicated for 20 min to get the dispersion solution. Subsequently, 255 μL SerMA solution (15%, w/v), 30 μL Irgacure 2959 (1%, w/v) and 15 μL GO dispersion solution (4 mg/mL) were mixed at room temperature. After that, the mixture solution was exposed under UV light (6 mW/cm^2^, 365 nm, Landun UV lamp) for 4 min to form SMH/GO composite hydrogel.

### 2.6 Characterization of hydrogel

FTIR spectra were measured by Spectrum One spectrometer (PerkinElmer). The morphology of lyophilized SMH/GO hydrogel was observed by scanning electron microscope (SEM, JSM-5610LV). In brief, lyophilized scaffolds were sputter-coated with gold and observed using scanning electron microscopy under high vacuum.

### 2.7 Degradation *in vitro*

The degradation ratios of SMH/GO hydrogels were calculated by weight loss in PBS (pH 7.4). Briefly, 200 μL SMH/GO hydrogel was immersed in PBS (2 mL, pH 7.4) and shaken slowly at 37 ^o^C. At Day 1, 2, 3, 5, 7, 10, 13, 24, 32 and 45, respectively, the supernatant was taken out. The SMH/GO hydrogels were lyophilized. The weight of SMH/GOs was recorded at given time points and considered as W_t_. The degradation ratio was calculated using the following equation: Degradation ratio (%) = (W_0_-W_t_)/W_0_×100% (W_0_ was the initial dry weight of the hydrogel and W_t_ was the dry weight of the hydrogel at different time points).

### 2.8 The mechanical property of SMH/GO hydrogels

The compressive stress of SMH/GO hydrogel was detected using Rheostress 6000 (Thermo Scientific, Germany) in a press mode. Briefly, 200 µL SMH/GO hydrogel was placed in the lower plate of the rheometer and compressed at a speed of 5 mm/min. The compressive force was recorded until SMH/GO hydrogel was deformed by upper plate.

### 2.9 Photoluminescent property

The lyophilized SMH/GO hydrogel was observed by a confocal laser-scanning microscope (Nikon A1Si, Japan). The emission spectra of SMH/GO hydrogel and SMH hydrogel were analyzed by a RF-5301 PC fluoro spectrophotometer (Shimadzu) with 320-600 nm wavelength. For *in vivo* imaging analysis, 200 μL SerMA/GO composite solution containing Irgacure 2959 (1 wt%, 10 μL) was subcutaneously injected into the dorsal skin of C57BL/6 mice (6 weeks old; Beijing Vital River Laboratory Animal Technology Co., Ltd.), and exposed under the UV light (6 mW/cm^2^) for 4 min to form SMH/GO hydrogel. The mice were observed using In-Vivo FX PRO (Bruker, Belgium) 2 hours later.

### 2.10 Establishment of rat calvarial defect model

SD rats (8 weeks old; Beijing Vital River Laboratory Animal Technology Co., Ltd.) were anesthetized and circular defect with the diameter of 4 mm on the right region of skull was created. After that, rats were divided into four groups randomly: (1) wounds without any treatment; (2) wounds covered by SMH hydrogel; (3) wounds treated with Osteobone; (4) wounds treated with SMH/GO-2 hydrogel. All the animal experiments were performed according to the guidelines for the care and use of laboratory animals (ethics committee of Tongji Medical College, Huazhong University of Science and Technology) and animal protocols were approved by the animal care and use committee of Huazhong University of Science and Technology, Wuhan, China.

### 2.11 X ray and MicroCT analysis

4, 8 and 12 weeks after treatments, rats were sacrificed and calvarias were taken out. The samples were observed by digital microradiography using In-Vivo FX PRO. The X-ray energy levels were 35 kV and 397 μA. The filter of 0.4 mm and the exposure time of 30 seconds were used for all the samples. The samples were also observed using microCT scanning (65 kV; Skyscan 1176, Bruker-microct, Belgium) at a resolution of 9 μm. 3D images of samples were measured using CTVox software (Skyscan Company). BV/TV (bone volume to total volume) and BS/TV (bone surface density) samples was calculated using CTAn software (Skyscan Company).

### 2.12 Histological analysis

At specific time intervals (4, 8, 12 weeks), the rats were sacrificed and regenerated tissues were carefully removed and fixed by 4% paraformaldehyde. Then, the samples were incubated with 0.5M EDTA solution (pH 8.0) for decalcification. After 7 to 10 days, the samples were embedded in paraffin and sliced into 4-μm thick sections. Finally, the tissue sections were immunohistochemically stained (H&E, Masson's, Osteocalcin (OCN), Collagen I (COL I), Runx 2, CD44 and CD73), and observed using a fluorescence microscope (Olympus IX71, Japan).

### 2.13 Cell proliferation

Bone marrow-derived mesenchymal stem cells (BMSCs) from rats' marrow were isolated as previously described 37. Briefly, SD (Sprague-Dawley) rats (6-week old) were sacrificed; femurs and tibias were isolated. Then, soft tissues were separated from femurs and tibias. After that, femurs and tibias were immersed in 75% ethanol. Five min later, the bone marrow cells were flushed out from femurs and tibias using a 10-mL syringe and suspended in 20 mL IMDM (Hyclone) medium. The cell suspension was filtered through a 70-μm filter to remove the bulk tissue. Then, cells were cultured in 10-cm culture dishes with IMDM medium containing 10% FBS. After 24 hours, the non-adherent cells were removed by replacing fresh IMDM medium. Finally, BMSCs were obtained and cultured in the dishes with IMDM containing 10% FBS.

BMSCs were seeded in 96 well plates at the density of 6000 cells/well. After 24 hours, the medium was replaced by serum-free medium supplement with SerMA/GO (0.2%, 0.4%, 0.8%, 1.0%, w/v). The cell viability was detected by MTT assay after incubation for 24 hours. The cell proliferation ratios were calculated using the following equation: Cell proliferation (%) = OD _SerMA/GO_/OD _original_× 100%, where OD_original_ is the absorbance measured for cells co-cultured with SerMA/GO for 4 hours and OD _SerMA/GO_ is the absorbance measured for cells co-cultured with SerMA/GO for 24 hours.

### 2.14 Cell migration assay

BMSCs were seeded in 6-well plates at the density of 1×10^5^ cells/well. After 24 hours, cells in the centers of the wells were removed using a cell scraper. Then, 200 µL SMH /GO hydrogel was placed in center of the wells. Cell migration was observed by microscopy and analyzed using Image J software after 24 hours.

Transwell assay of BMSCs was detected using 24-well Boyden chambers (24 mm diameter, 8 μm pores, Corning, NY, USA). Briefly, BMSCs were seeded on the upper chamber of with serum-free IMDM medium, and IMDM medium containing 200 µL SMH/GO hydrogel was added in the lower chamber. The cells were incubated at 37 ^o^C for 24 hours and stained with crystal violet. The representative images were taken by microscopy.

### 2.15 Osteogenic differentiation

BMSCs were seeded in 6-well plates with a density of 2×10^5^ cells/ well at 37 ^o^C for 24 hours. Subsequently, cells were treated with fresh IMDM medium containing SMH and SMH/GO for 24 hours and measured by immunofluorescent stain (Ocn, Col I and Runx 2). The total mRNA was isolated with Trizol reagent (Invotrigen) and reversely transcribed to cDNA. Quantification of cDNA was assessed using quantitative Real-Time PCR with the SYBR Green I Assay (Takara). The primers are listed in **Table S1.**

### 2.16 RNA sequencing and analysis

BMSCs were treated with SMH/GO for 24 hours and the total RNA was extracted by Trizol reagent. Sequencing libraries were generated using NEBNext®UltraTM RNA Library Prep Kit following manufacturer's recommendations and sequenced by Illumina Hiseq X-ten platform. The generated reads were mapped to the reference genome using Hisat2 v2.0.5 software and quantificated by featureCounts v1.5.0-p3 software. Differential expression analysis was performed using the DESeq2 R package. Gene Ontology (GO) enrichment and Kyoto Encyclopedia of Genes and Genomes (KEGG) analysis were performed using cluster Profiler R package. Gene Set Enrichment Analysis (GSEA) was performed.

### 2.17 Statistical Analysis

All the data was expressed as mean ± standard deviation. Statistically significant differences between groups were evaluated using one-way analysis of variance (ANOVA) at a confidence interval of 95%.

## 3. Results

### 3.1 Preparation and characterizations of SMH/GO hydrogels

**Figure 1:**
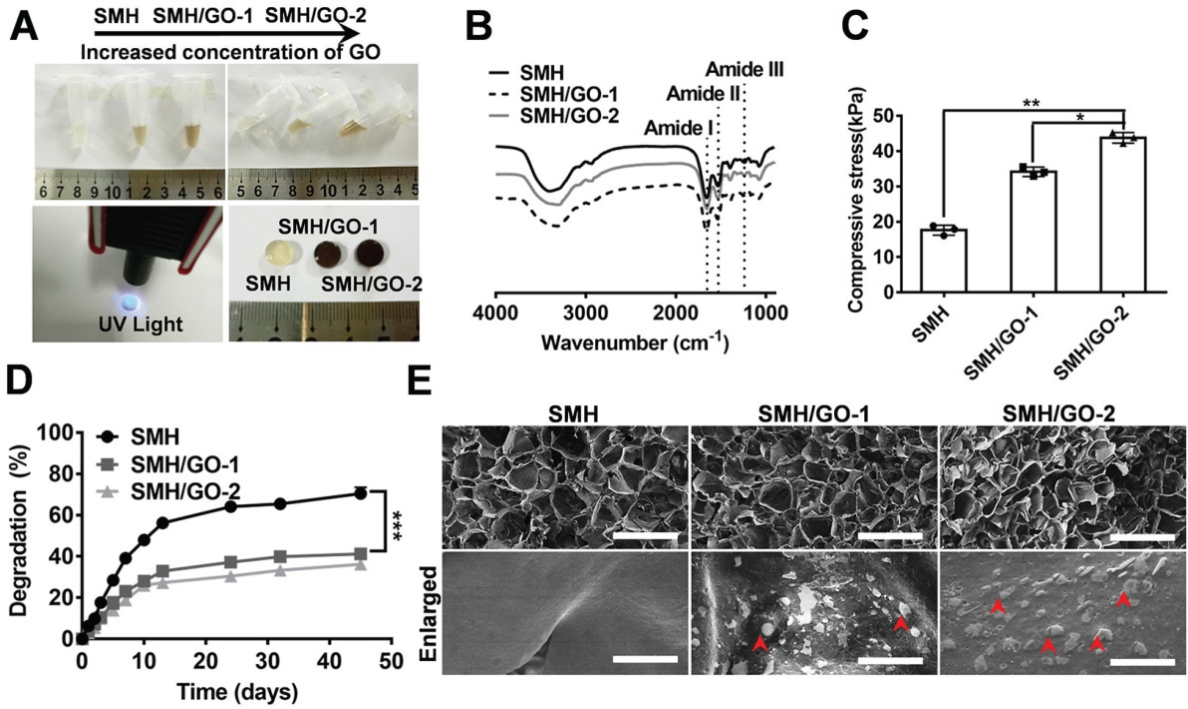
** Characterizations of SMH/GO hydrogels.** (A) Preparation of SMH/GO hydrogels via UV light at 365 nm. (B) FTIR spectra of SMH/GO hydrogels. (C) Mechanical properties of SMH/GO hydrogels. (D) Degradation profiles of SMH/GO hydrogels in PBS (pH7.4, 37 ^o^C). (E) Scanning electron micrographs of SMH (left), SMH/GO-1 (middle) and SMH/GO-2 (right). Scale Bars, 500 μm (upper panel) and 10 μm (lower panel). Red arrowheads indicate graphene oxide, *P<0.05, **P<0.01, ***P<0.001. n = 3 per group per condition.

Ideal artificial bone substitutes are expected to possess suitable mechanical strength for fulfilling the supporting function of the bone 38. Previously, we prepared a photo-crosslinked sericin methacryloyl hydrogel (SMH) 25. However, the relatively weak mechanical property of this SMH hardly meets the demands for bone repair. To improve its mechanical property and enhance its osteogenic induction ability, methacrylated sericin (SerMA) solution was mixed with different concentrations of graphene oxide (GO) (0.02% for SMH/GO-1 and 0.04% for SMH/GO-2) (**Table 1**) and exposed to UV light (6mW/cm^2^) for crosslinking to form SMH/GO composite hydrogels (SMH/GO) (**Figure 1A**). FTIR analysis on SMH/GO hydrogels indicated that the amide I of SMH at 1658 cm^-1^ was close to 1660 cm^-1^ for SMH/GO-1 and 1655 cm^-1^ for SMH/GO-2, suggesting the addition of GO does not cause secondary structural changes (**Figure 1B**). As expected, the addition of GO significantly enhanced the mechanical property of these hydrogels in a GO-concentration dependent manner (**Figure 1C**). The compressive moduli were 17 kPa for SMH, 34 kPa for SMH/GO-1, and 43 kPa for SMH/GO-2 (**Figure 1C**). The degradation rates of these hydrogels were negatively correlated with GO contents (degradation rates: 73% for SMH, 43% for SMH/GO-1, and 39% for SMH/GO-2 on Day 45) (**Figure 1D**). The lyophilized SMH/GO hydrogels were highly porous with GO particles clearly observed on the surface and interior of the hydrogels (**Figure 1E**). The SMH/GO hydrogels inherited sericin's fluorescent properties with the emission spectra falling between 300-600 nm wavelength (**Figure S1B**). The lyophilized SMH/GO hydrogels could be visualized under the excitation light at 405 nm for blue, 488 nm for green, and 561 nm for red fluorescence (**Figure S1A**). Of note, these hydrogels could be still visualized underneath of the dorsal skin of the mice by their photoluminescence (**Figure S1C**). Furthermore, SerMA/GO (precursor solution of SMH/GO hydrogels) could promote the proliferation of BMSCs in serum-free medium in a dose dependent manner (**Figure S2**). The proliferation rate of BMSCs cultured in serum-free medium containing 1.0 % (w/v) SerMA/GO was similar to that of the cells cultured in the medium containing 10% fetal bovine serum (FBS) (**Figure S2**). This was largely due to sericin's known cell-nutritious effect, consistent with previous studies 25, and this result also indicates the good biocompatibility of SMH/GO hydrogels.

### 3.2 SMH/GO hydrogels as an artificial bone substitute accelerate bone regeneration of calvarial defects

**Figure 2:**
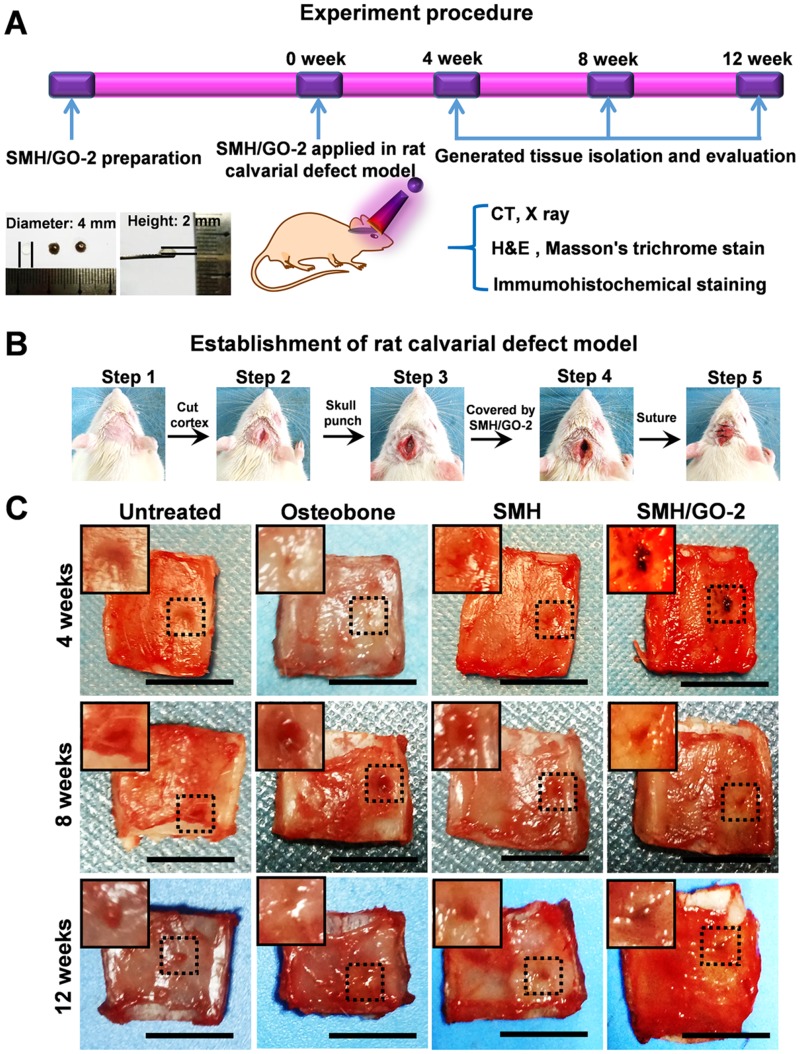
** SMH/GO hydrogels as an artificial bone substitute for bone regeneration after calvarial defect*.*** (A) The experimental procedure using SMH/GO hydrogels for rat calvarial defect treatment. (B) The establishment of the rat calvarial defect model. (C) Photographs of the wounds in the animals receiving no treatment (control), Osteobone, SMH, and SMH/GO-2 treatments, respectively. Scale bars, 1 cm. n = 6 per group per condition.

Adequate mechanical strength is key to the design and fabrication of ideal artificial bone substitutes 38. Compared to SMH/GO-1 (34 kPa), SMH/GO-2 (43 kPa) had the higher compressive stress and was close to that of the normal bone tissue 38. Thus, SMH/GO-2 was selected as an artificial bone substitute for bone regeneration after calvarial defects and used for subsequent studies (**Figure 2A, B**). Osteobone, a commercial inorganic-element-based artificial bone, was chosen as the control. Twelve weeks after treatment, bone regeneration in the rats receiving the SMH/GO-2 treatment was significantly more effective than the untreated group and the SMH-only group and comparable to the rats receiving Osteobone treatment (**Figure 2C**). These observations indicate that SMH/GO hydrogels effectively promote bone regeneration.

### 3.3 Radiological evaluation after treatment

**Figure 3:**
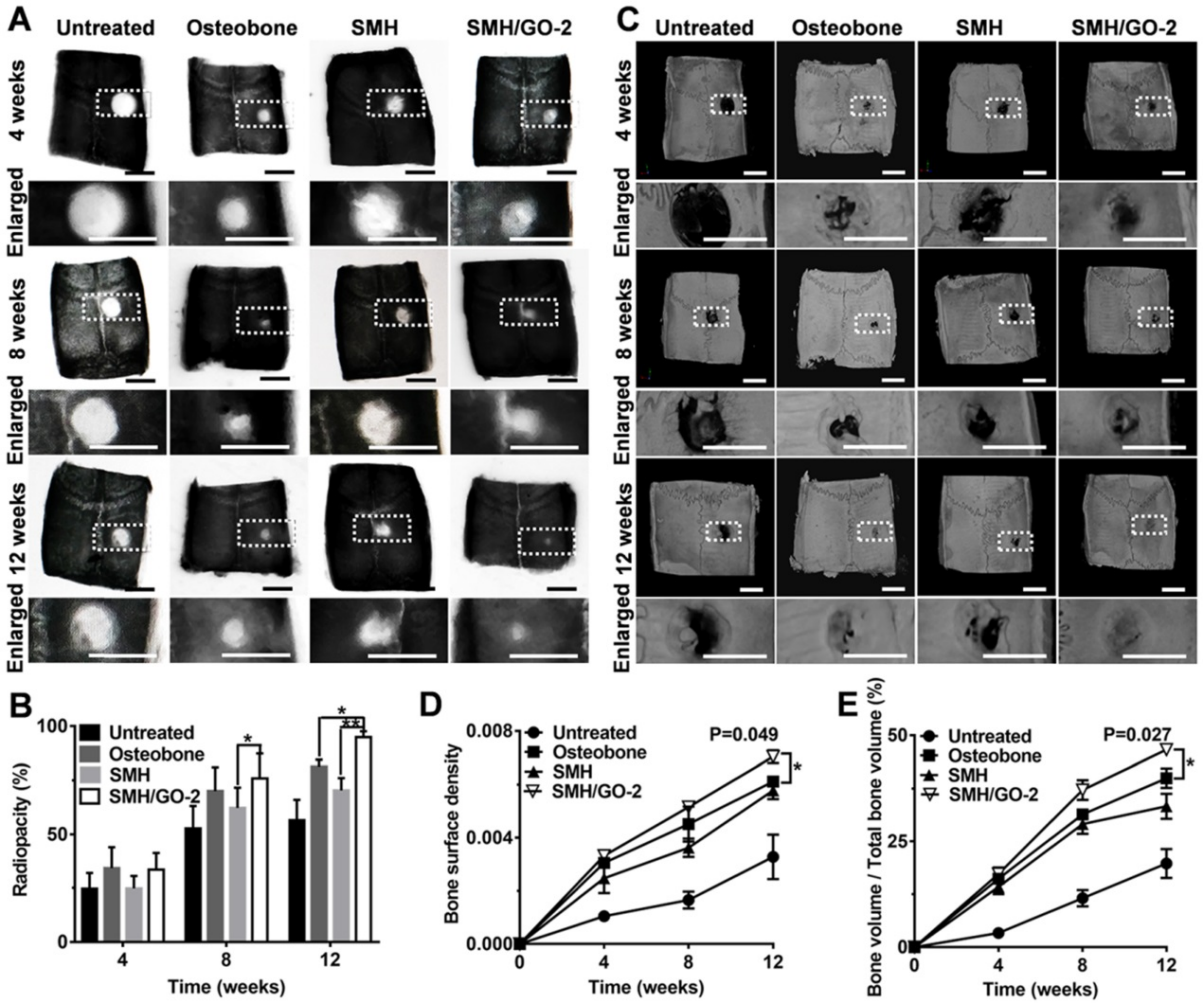
** Radiological evaluation of bone regeneration after treatment.** (A) Radiographs of defective area 4, 8, 12 weeks after treatment. (B) Radiopacity of calvarial defects 4, 8, 12 weeks after treatment was quantified using Image J. (C) MicroCT reconstruction of defective area 4, 8, 12 weeks after treatment. (D-E) Micromorphometric bone parameters of the calvarial defects after treatment including bone surface density (bone surface area of regenerated tissue (µm^2^) / total bone volume of wound site (µm^3^)) (D) and relative bone volume (bone volume of regenerated tissue/ total bone volume of wound site×100%) (E). *P<0.05, ** P<0.01; n=6 per group per condition. Scale bars, 5 mm.

To further assess bone regeneration, the defect sites at Week 4, 8 and 12 after treatment was examined by X-ray and MicroCT imaging. The analysis of radiopacity in defective area of calvarial bone showed that SMH/GO-2 hydrogel treatment resulted in the most effective new bone regeneration (SMH/GO-2, 94.7%; Osteobone, 81.2%; SMH, 70.3%; untreated, 56.5%) during 12 weeks after treatment (**Figure 3A, B**). The similar regenerative outcomes across these treatments were also observed by MicroCT imaging (**Figure 3C**). The bone surface density of SMH/GO-2 group (0.007) and the relative bone volume of SMH/GO-2 group (46.6%) were significantly higher than the Osteobone treatment group (0.006 for bone surface density; 39.9% for relative bone volume) 12 weeks after treatment (**Figure 3D, E**). Together, these results demonstrate that SMH/GO hydrogels effectively accelerate bone regeneration after bone injury.

### 3.4 Histological evaluation for bone regeneration

**Figure 4:**
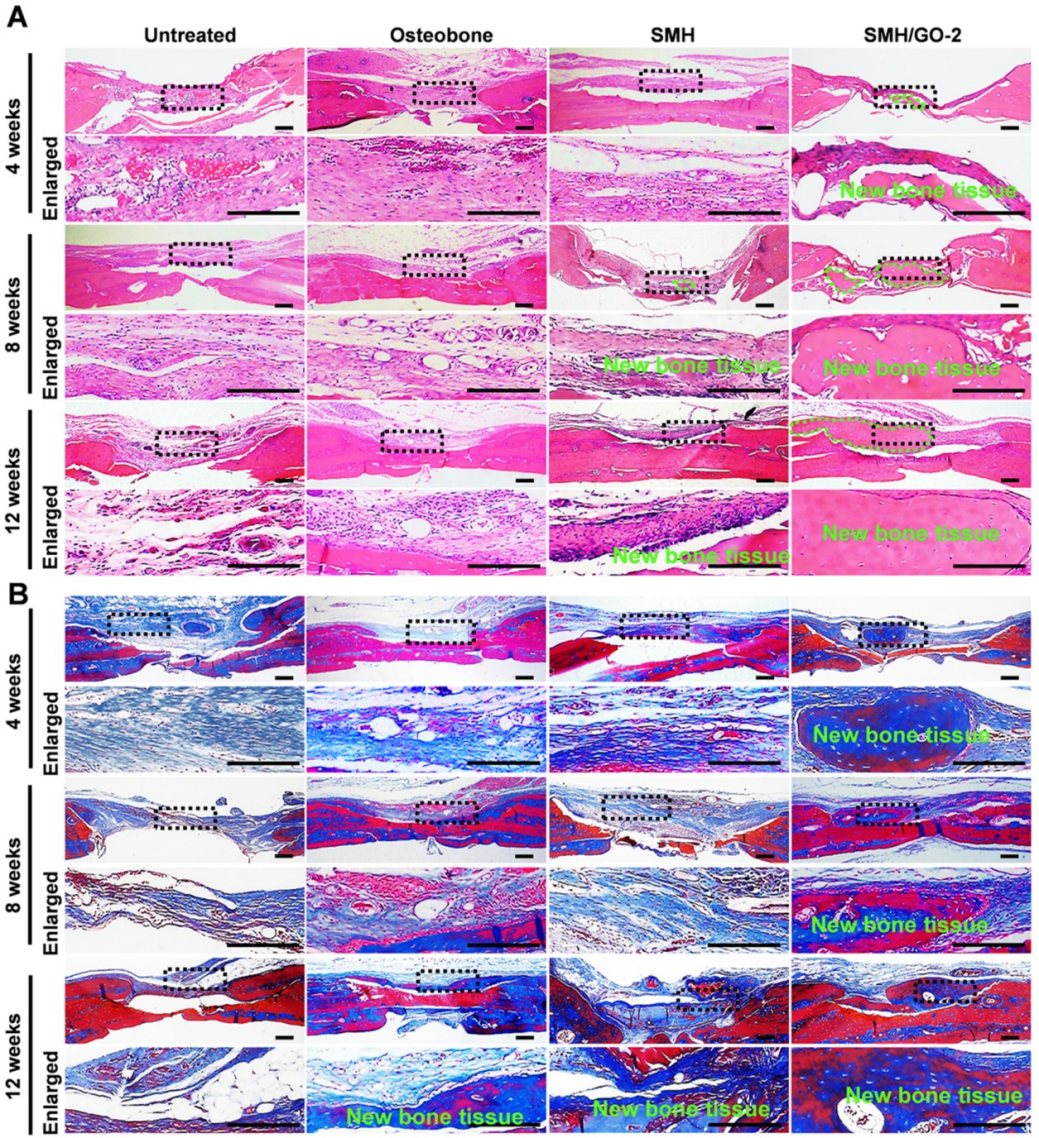
** Histological evaluation of bone regeneration after treatment.** (A) Hematoxylin-eosin (H&E) and (B) Masson's trichrome staining of newly generated bone tissue 4, 8 and 12 weeks after treatments. The black dotted boxes in the upper panels were enlarged in the lower panels. The area marked by the dotted green line in (A) was new bone tissue. n=6 per group. Scale bars, 200 μm.

To histologically investigate bone regeneration, the regenerated tissues at defect sites were isolated and examined using hematoxylin and eosin (H&E) (**Figure 4A**) and Masson's (**Figure 4B**) staining for observing new bone tissue formation at Week 4, 8 and 12 after treatment. Compared to the control, bone-like tissue was visually observed 4 weeks after implantation and the newly regenerated bone area progressively increased after 8 and 12 weeks in the SMH/GO-2 treated group, suggesting that SMH/GO hydrogels promote remodeling of the bone structure after injury. Taken together, these observations indicate that SMH/GO hydrogels can serve as a suitable artificial bone substitute and enable effective histological bone regeneration.

### 3.5 Osteogenesis induced by SMH/GO treatment after bone injury

**Figure 5:**
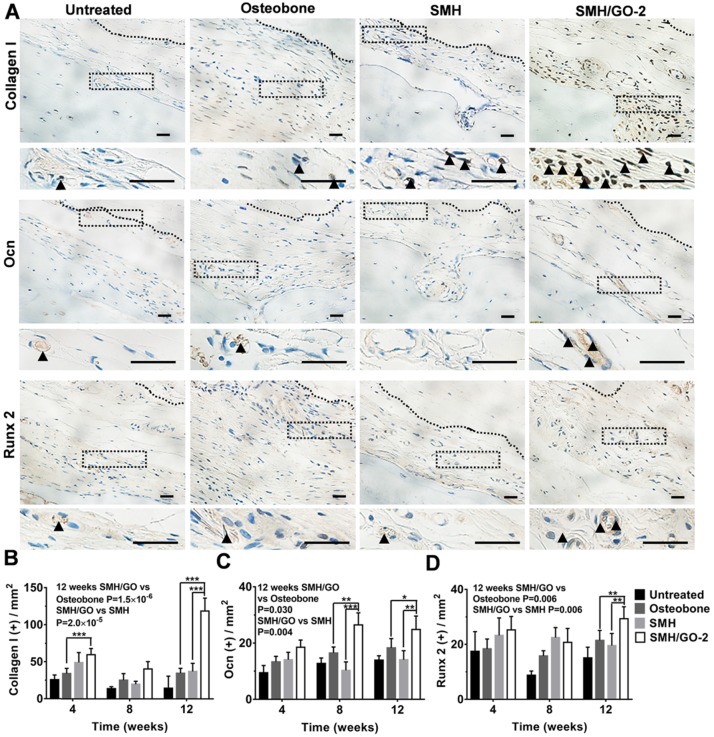
** Osteoblasts in wound site after SMH/GO hydrogel's treatment.** (A) The immunohistological staining of Collagen I, Ocn and Runx 2 at the wound sites at 12 weeks after treatment. The black dotted boxes in the upper panels were enlarged in the lower panels. The black triangle indicates positively stained cells. (B-D) Quantification of the numbers of Collagen I (B), Ocn (C) and Runx 2 (D) positive cells in (A) and Fig. S3. *P<0.05, **P<0.01, ***P<0.001. n=6 per group per condition, six random fields per slide and 3 slides per animal. Scale bars, 20μm.

Osteoblasts are crucial for osteogenesis and bone remodeling. To assess whether SMH/GO impacted osteoblasts, the regenerated tissues were immumohistochemically stained by osteoblast markers (Collagen I, Ocn and Runx 2) 4, 8 and 12 weeks after treatment, respectively. The number of cells stained positively for Collagen I in the wounds treated with SMH/GO-2 hydrogels (59 cells/cm^2^) were nearly 50% more than the Osteobone group (34 cells/cm^2^) after 4 weeks (**Figure 5A, B** and **Figure S3**). Moreover, after 12 weeks, the number of osteoblasts in the wound region treated with SMH/GO-2 hydrogels (25 cells/cm^2^ for Ocn^+^,30 cells/cm^2^ for Runx 2^+^, and 119 cells/cm^2^ for Collagen I^+^) were significantly more than those treated with Osteobone group (18 cells/cm^2^ for Ocn^+^, 22 cells/cm^2^ for Runx 2^+^ and 35 cells/cm^2^ for Collagen I ^+^) and SMH (14 cells/cm^2^ for Ocn^+^, 20 cells/cm^2^ for Runx 2^+^ and 37 cells/cm^2^ for Collagen I ^+^, respectively) (**Figure 5A-D**). Together, these results provide direct evidence demonstrating that SMH/GO hydrogels significantly increase the quantity of osteoblasts both at the early and the late stage of bone regeneration.

### 3.6 SMH/GO hydrogels accelerate osteogenesis by inducing BMSCs migration and osteogenesis differentiation

**Figure 6:**
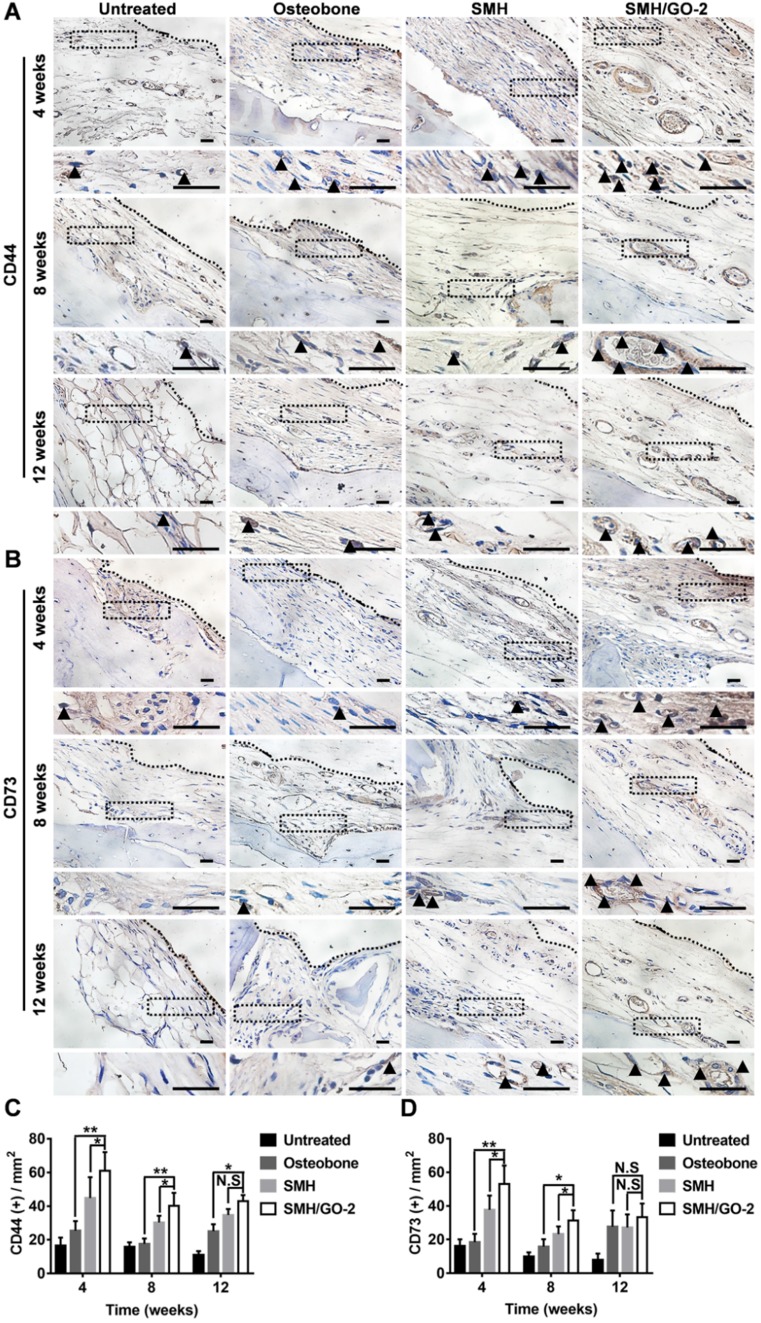
** BMSCs in wound sites after treatments.** The immunohistological staining of (A) CD44 (BMSCs marker) and (B) CD73 (BMSCs marker) in wound site 4, 8 and 12 weeks after treatments. The black dotted boxes in the upper panels were enlarged in the lower panels. The black triangles indicate positive cells. (C) Quantification of CD44 positive cells in (A). (D) Quantification of CD73 positive cells in (B). *P<0.05, **P<0.01, N.S, not significant. n=6 animal per group per condition, six random fields per slide and 3 slides per animal. Scale bars, 20μm.

BMSCs are a type of pluripotent stem cells with the ability of differentiating into osteoblasts, myoblasts and adipocytes under certain conditions 39, 40, and play an important role in bone formation after injury. In previous study, sericin or its derivative was found to promote direct migration of BMSCs to the sites of implantation 28. GO was previously reported to have osteogenic induction property 31. Therefore, we assessed whether the increased number of osteoblasts at the wound area treated with SMH/GO hydrogels (**Figure 5**) was attributed to BMSCs recruitment and their osteogenic differentiation. The tissues were stained for BMSCs' markers, CD44, CD73, at Week 4, 8 and 12 after treatment (**Figure 6A, B**). The number of BMSCs in the SMH/GO-2 hydrogel treated group (60 cells/mm^2^ for CD44^+^, 53 cells/mm^2^ for CD73^+^) were approximately 1.5-fold higher than SMH group (44 cells/mm^2^ for CD44^+^, 37 cells/mm^2^ for CD73^+^) (**Figure 6C, D**) and 2.5-fold higher than Osteobone group (25 cells/mm^2^ for CD44^+^, 18 cells/mm^2^ for CD73^+^) (**Figure 6C, D**). To determine whether the accumulation of BMSCs was due to migration-promoting functions of SMH/GO hydrogels, the effects of SMH/GO hydrogels on BMSCs migration were investigated by wound closure assay (**Figure S4A**) and transwell assay (**Figure S4D**). After 24-hour treatment, the number of BMSCs that migrated towards SMH/GO-2 hydrogels (254 cells/mm^2^) were significantly higher than the SMH group (211 cells/mm^2^) and the untreated (118 cells/mm^2^) group (**Figure S4A-C**). The results of transwell assays were also similar and consistent with wound closure assay (**Figure S4D, E**). Overall, the SMH/GO hydrogel promotes BMSCs migration towards the hydrogel at the implantation site.

**Figure 7:**
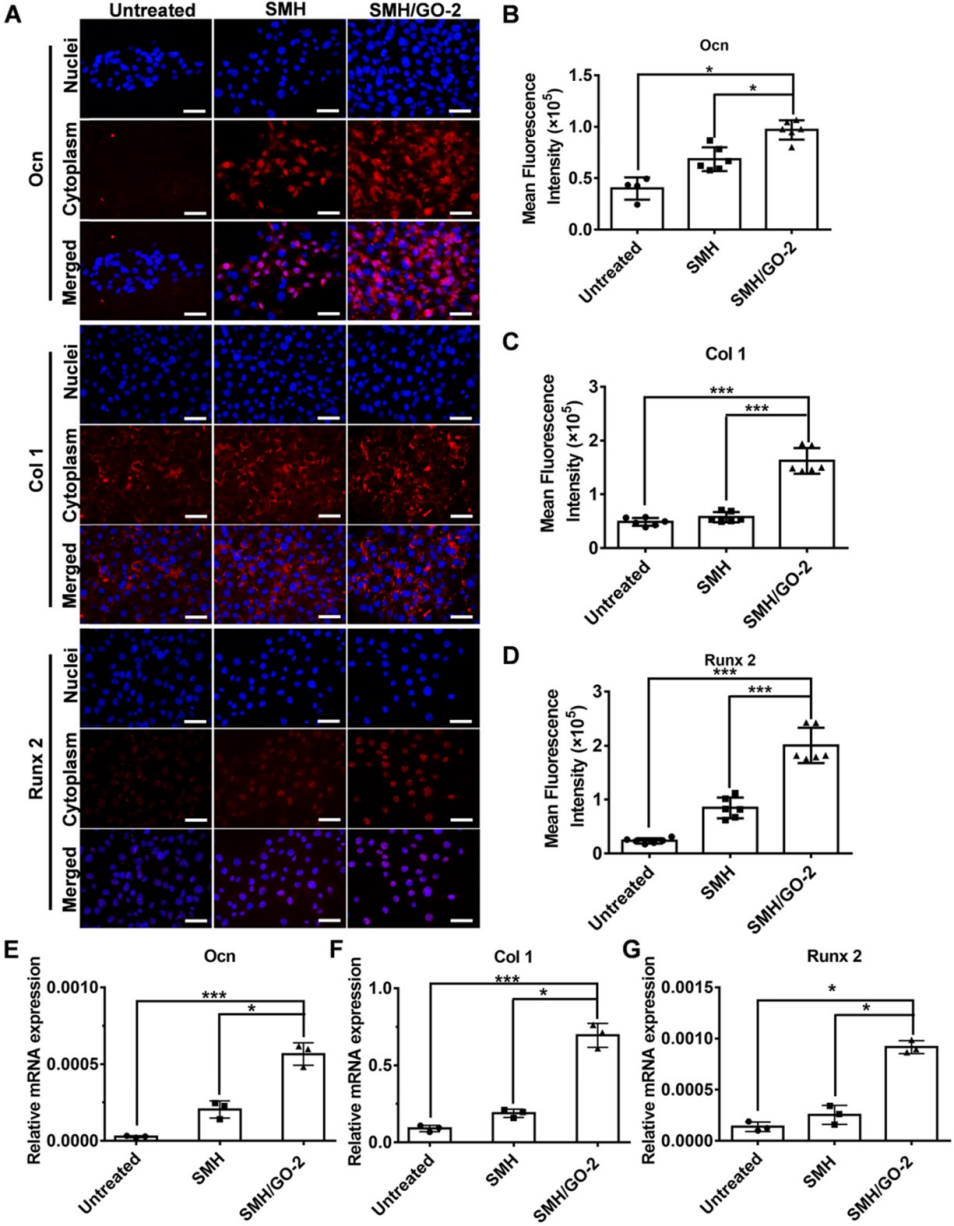
** BMSCs osteogenesis differentiation after treatment.** (A) BMSCs were immunofluorescently stained with (Ocn, Col 1 and Runx 2) after being co-cultured with SMH/GO-2 or SMH hydrogels for24 hours. (B-D) Quantification of red fluorescence intensity of BMSCs in (A). (E-G) The relative mRNA expression of three osteogenesis genes (Ocn (E), Col1 (F) and Runx 2(G)) in BMSCs co-cultured with SMH/GO-2 or SMH hydrogels for 24 hours. *P<0.05, ***P<0.001, N.S: not significant. n=3 per group. Three random fields per group were quantified. Scale bars, 50 μm.

We then assessed whether SMH/GO hydrogels had effects on osteogenic differentiation of BMSCs. BMSCs were immunofluorescently stained for the well-known osteogenic markers, Ocn, Col 1 and Runx 2, after being co-cultured with SMH/GO-2 hydrogels for 24 hours. The protein expression of Ocn, Col 1 and Runx 2 in the SMH/GO-2 treated group was significantly higher than the SMH and the untreated group (**Figure 7A-D**). Consistently, SMH/GO-2 hydrogels markedly upregulated the mRNA levels of Ocn, Col 1 and Runx 2 in BMSCs (**Figure 7E-G**). Together, these results suggest that SMH/GO hydrogels accelerate bone regeneration after injury likely through mediating BMSCs directional migration and osteogenesis differentiation.

### 3.7 The mechanisms of SMH/GO hydrogels regulating BMSCs migration and osteogenic differentiation

**Figure 8:**
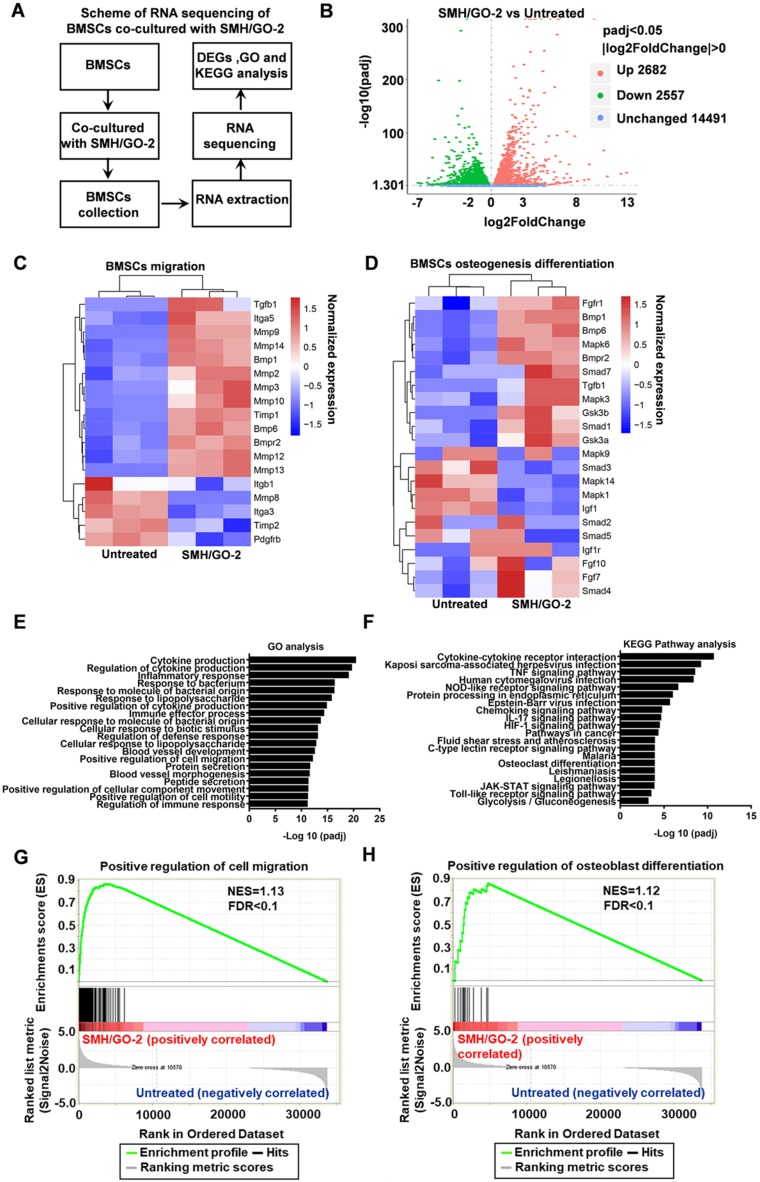
** RNA sequencing of BMSCs co-cultured with SMH/GO hydrogels.** (A) The scheme of RNA sequencing of BMSCs co-cultured with SMH/GO-2 hydrogel. (B) Volcano plots for all the genes of the SMH/GO-2 hydrogel group compared with those of the untreated group. The red and green dots indicate up- and down-regulated DEGs with padj< 0.05, and blue dots indicate unchanged genes. (C-D) Heat map of genes associated with BMSCs migration (C) and osteogenic differentiation (D) in the SMH/GO-2 hydrogel group and the untreated group. (E) Top 20 GO terms associated with biological processes (padj<0.05) involving up-regulated genes in the SMH/GO-2 hydrogel group. (F) Top 20 enriched KEGG pathways with up-regulated genes (padj<0.05) in the SMH/GO-2 hydrogel group. (G-H) Gene Set Enrichment Analysis (GSEA) of gene sets for positive regulation of cell migration (G) and positive regulation of osteoblast differentiation (H). NES, normalized enrichment score. FDR, false discovery rate. Three samples were measured per group.

To investigate the potential mechanisms of SMH/GO hydrogels regulating BMSCs migration and differentiation, BMSCs treated with SMH/GO-2 hydrogels were subjected to transcriptomics RNA sequencing (**Figure 8A**) with the untreated BMSCs as the control. The analysis of differentially expressed genes (DEGs) revealed that the expression of 2682 genes were up-regulated and 2557 genes were down-regulated in the SMH/GO-2 hydrogel group (p-value adjusted for multiple testing (padj) <0.05) compared to the untreated group (**Figure 8B**).

The expression of the key genes related to BMSC cell migration (such as BMP6, TGF-β1, MMP2, MMP3, MMP8, MMP10, and MMP12) (**Figure 8C**) and osteogenic differentiation (such as TGF-β1, Smad1, BMP6, FGF7, MAPK3, MAPK6 and GS3K) (**Figure 8D**) were higher in the SMH/GO-2 hydrogel group than those in the untreated group. To better illuminate the functions of these differentially expressed genes, the enrichment analysis for Gene Ontology (GO) terms was conducted for up-regulated genes. These results suggest that SMH/GO hydrogels promote BMSCs migration and osteogenesis differentiation likely through regulating the biological processes involved in “Positive regulation of cell migration”, “Positive regulation of osteoblast differentiation” and “Positive regulation of myeloid cell differentiation” (**Figure 8E** and **Table 2**). Among the pathways enriched with up-regulated genes in the SMH/GO-2 hydrogel treated group, we identified three critical signaling pathways associated with BMSCs migration and osteogenesis differentiation by KEGG pathway analysis, including: “MAPK signaling pathway”, “TNF signaling pathway”, and “Chemokine signaling pathway” (**Figure 8F**, **Table 3**,**Table S2**,**Table S3** and**Table S4**). Consistently, we found that genes related to cell migration and osteoblast differentiation were enriched significantly in the SMH/GO hydrogel treated BMSCs by GSEA analysis (**Figure 8G, H**). Taken together, these data suggest that SMH/GO hydrogels may facilitate BMSCs migration and osteogenic differentiation via modulating the aforementioned three major signaling pathways.

## 4. Discussion

Severe bone defects cannot heal by itself and requires artificial bone substitutes for treatment. Currently, the most commonly used clinical bone substitutes are mainly composed of titanium alloy and other metals, which are often challenged by slow degradation rate and limited biocompatibility 10. During bone repair, another major obstacle for successful bone regeneration is the availability of responsive bone-forming progenitor cells, which can be recruited to the wound site, proliferate, and eventually differentiate into bone cells.

BMSCs are the main type of progenitors cells involved in the bone formation process. After injury, BMSCs are recruited to the site of injury by chemokines and differentiate into osteoblasts and osteoclasts. Thus, recruitment and osteogenic differentiation of BMSCs are the key factors for effective bone regeneration. However, most of the currently available biomaterials only have the filling functions and lack osteogenic induction properties. Although the delivery of exogenous BMSCs via biomaterials is effective for bone healing, it is limited by immune rejection and unsuitable for bone repair that often takes a relatively long time. Previously, porous calcium phosphate (CaP) bioceramics reportedly possessed certain osteoinduction property, possibly due to the fact that microporous structure could absorb the endogenous bone growth factors and stimulate the BMSCs differentiation. However, the osteo-induction mechanism of the bioceramics remains unclear and they may induce immune rejection 18. In addition, a variety of commercial artificial bone substitutes have been widely used in treating bone defects. Osteobone, a commercial artificial bone composed of calcium, phosphorus and silicon, exhibits ideal properties as an artificial bone substitute with suitable porosity, osteoconductive property and appropriate mechanical strength. Bio-Oss®, a deproteinized bovine bone, has been applied in clinical bone transplantation due to its osteoconductive activity. Nevertheless, Bio-Oss® is extracted from cancellous bone, which makes it not optimal for repairing dense bone defects 41. Therefore, development of biocompatible and biodegradable artificial bone substitutes capable of BMSCs recruitment and osteogenic differentiation is highly desired.

Sericin is a natural protein, exhibiting low immunogenicity, biodegradation, cell adhesive property, cell proliferation and migration promoting effects 42, 43. Although sericin has been successfully used in regeneration of sciatic nerve 44, myocardial tissue 45 and cartilage tissue 25, it has never been used for bone regeneration, mainly due to fact that the sericin lacks sufficiently high mechanical strength that are required for bone repair. Given that sericin was able to mediate BMSCs migration revealed by our previous studies 28, sericin may be used as an artificial bone substitute for severe bone defects if its mechanical and osteo-induction properties could be improved and acquired. Therefore, GO, an inexpensive, non-toxic material that has excellent mechanical property and osteogenic property 46-48, was used in combination with sericin hydrogels to form a SMH/GO composite hydrogels in our study. The bone regeneration in the SMH/GO hydrogel treated group was significantly faster than the Osteobone group and the SMH group at Week 12. Importantly, SMH/GO accelerated bone regeneration via mediating BMSCs migration and osteogenic differentiation. This SMH/GO-promoted BMSCs migration and osteogenic differentiation was tightly associated with the degradation products of hydrogels. Normally, BMSCs are recruited to wound sites by chemokines and pro-inflammatory cytokines at the early stage of bone healing 49. The recruited BMSCs mineralize (mineralized nodules formation is a typical marker for BMSCs osteogenic differentiation) and differentiate into osteoblasts in the presence of an array of cytokines, thus promoting bone repair. Of note, the degradation products of SMH/GO-2 mainly consisted of SMH's degradation products and grapheme oxide. Given that SMH degradation products were found to promote BMSCs migration in our previous study 28 and GO reportedly has osteogenic induction capability, SMH/GO hydrogel degradation products possibly serve as a cytokine analogue for mechanistically recruiting BMSCs and promoting BMSCs mineralization and differentiation into osteoblasts. By using RNA sequencing, the possible mechanism underlying SMH/GO hydrogels promoting bone regeneration was molecularly analyzed. SMH/GO hydrogel treatment upregulated the expression of BMSCs migration and osteogenic differentiation gene sets (e.g. BMP6, TGF-β1, MMP2, MMP3, MMP8, MMP10, MMP12 for migration and TGF-β1, Smad1, BMP6, FGF7, MAPK3, MAPK6, GS3K for osteogenesis differentiation). After being co-cultured with SMH/GO hydrogels, three main signaling pathways leading to BMSCs migration and osteogenesis differentiation were found to be activated, including: MAPK, TNF, and chemokine signaling pathways. Therefore, SMH/GO hydrogels can stimulate BMSCs migration and osteogenic differentiation by activating MAPK, TNF, and chemokine signaling pathways, resulting in effective bone regeneration.

## 5. Conclusion

We have designed and fabricated a photo-crosslinked SMH/GO composite hydrogel as an artificial bone substitute for effective bone repair in a rat calvarial defect model. This hydrogel inherited sericin's advantageous properties, such as good biocompatibility, biodegradation, cell adhesion, and cell-proliferation promoting effect. SMH/GO composite hydrogels possessed tunable mechanical properties for bone tissue regeneration. Importantly, this hydrogel is capable of facilitating osteogenesis via regulating autologous BMSCs migration and osteogenic differentiation. Thus, this study may provide a new type of effective, safe, and economical substitute for effective bone regeneration.

## Supplementary Material

Supplementary figures and tables.

## Figures and Tables

**Scheme 1:**
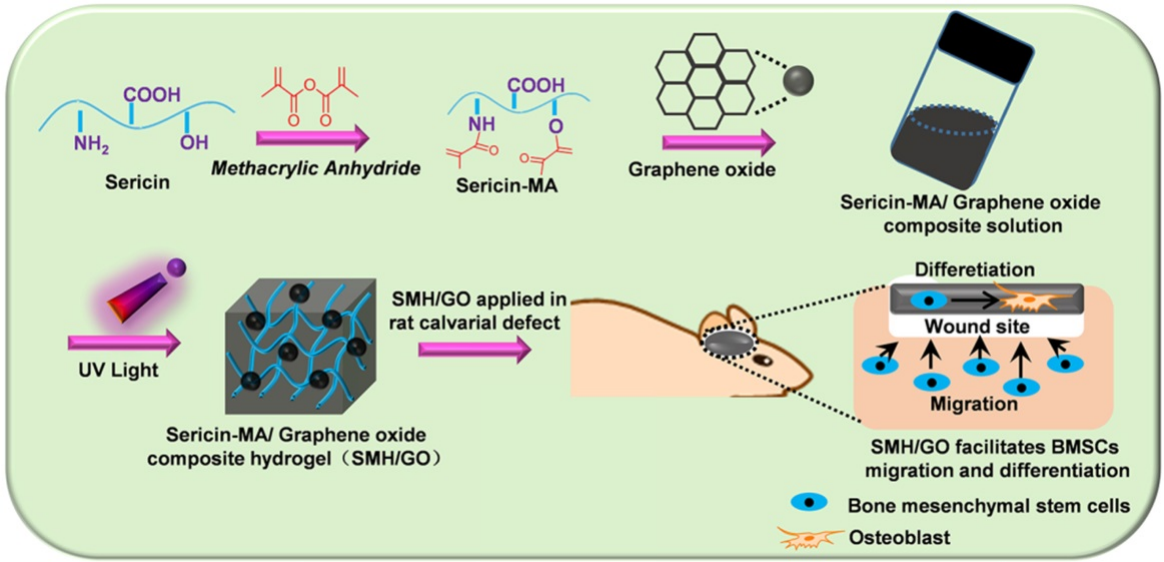
SMH/GO hydrogels as an artificial bone substitute for bone regeneration in a rat calvarial defect model via regulating BMSCs migration and osteogenesis differentiation.

**Table 1:** Components of SMH/GO hydrogels

Composite hydrogel	SMH	SMH/GO-1	SMH/GO-2
SerMA (wt %)	15	15	15
Graphene oxide (wt %)	0.0	0.02	0.04

**Table 2:** Up-regulated genes involved in the BMSCs migration and osteogenesis differentiation biological process (SMH/GO-2 vs Untreated).

Biological process	Gene numbers	padj
Positive regulation of cell migration	126	8.58E-13
Positive regulation of myeloid cell differentiation	23	6.11E-05
Positive regulation of osteoblast differentiation	16	0.044162

**Table 3:** Up-regulated genes involved in the BMSCs migration and osteogenic differentiation pathways (SMH/GO-2 vs Untreated).

KEGG pathway	Gene numbers	padj	Function
MAPK signaling pathway	70	0.015768	BMSCs migration and osteogenesis differentiation
TNF signaling pathway	50	3.28E-09	BMSCs migration
Chemokine signaling pathway	57	1.96E-05	BMSCs migration

## Abbreviations

MA: methacryloyl anhydride
GO: graphene oxide
SMH: sericin methacryloyl hydrogel
SMA: sericin methacyloyl anhydride
BMSCs: bone marrow-derived mesenchymal stem cells
MAPK: mitogen-activated protein kinase
TNF: tumor necrosis factor
BV: bone volume
TV: tissue volume
BS: bone surface
DMEM: Dulbecco's modified Eagle's medium
IMDM: Iscove's modified Dubecco's medium
FBS: fetal bovine serum
HE: hematoxylin & eosin
mRNAs: messenger RNAs
SEM: scanning electron microscopy
GO: gene ontology
KEGG: kyoto encyclopedia of genes and genomes
GSEA: gene set enrichment analysis
DEGs: differentially expressed genes
BMP: bone morphogenetic protein
MMP: matrix metalloprotein
TGF-β: transforming growth factor-β
CaP: calcium phosphate
NES: normalized enrichment score
FDR: false discovery rate
FPKM: Fragments Per Kilobase of transcript per Million fragments mapped
