# Supplementary material for: A sericin/ graphene oxide composite scaffold as a biomimetic extracellular matrix for structural and functional repair of calvarial bone
Source: Theranostics. 2020 Jan 1;10(2):741–56. doi: 10.7150/thno.39502 (PMC6929981; doi:10.7150/thno.39502)
Supplement: Supplementary file 1 — Supplementary figures and tables. [file thnov10p0741s1.pdf]

Supporting Information

A sericin/ graphene oxide composite scaffold as a biomimetic extracellular matrix for structural and functional repair of calvarial bone

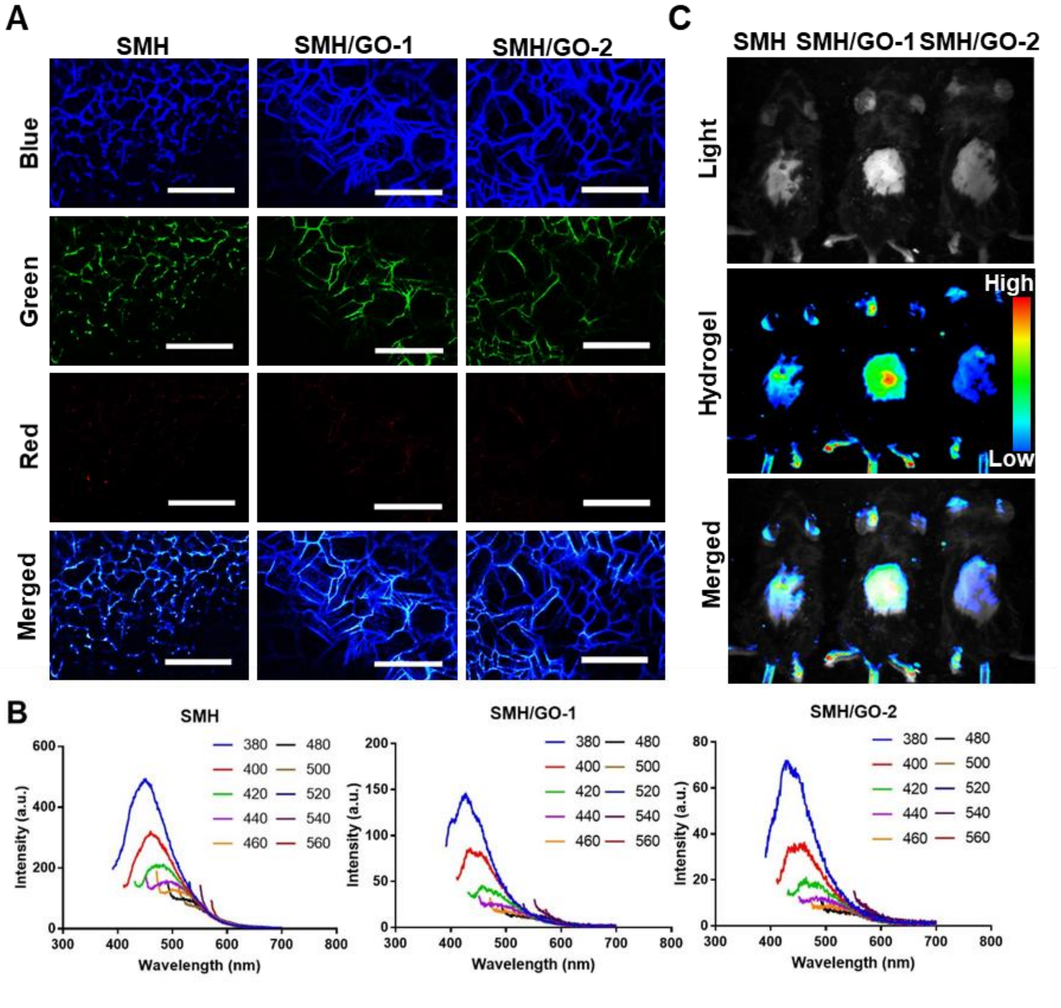

Figure S1. Photoluminescent property of SMH/GO hydrogels. (A) The lyophilized SMH, SMH/GO-1 and SMH/GO-2 were observed by confocal laser scanning microscopy under the exciting light with the wavelengths of 405 nm (Blue), 488 nm (Green) and 561 nm (Red). Scale bars, 200 μm. (B) The emission spectra of SMH, SMH/GO-1 and SMH/GO-2. (C) *In vivo* light and fluorescence images of SMH/GO hydrogels subcutaneously injected into the dorsal skin of C57BL/6 mice. Each picture was imaged under the excitation wavelength of 430 nm and the emission wavelength of 535 nm.

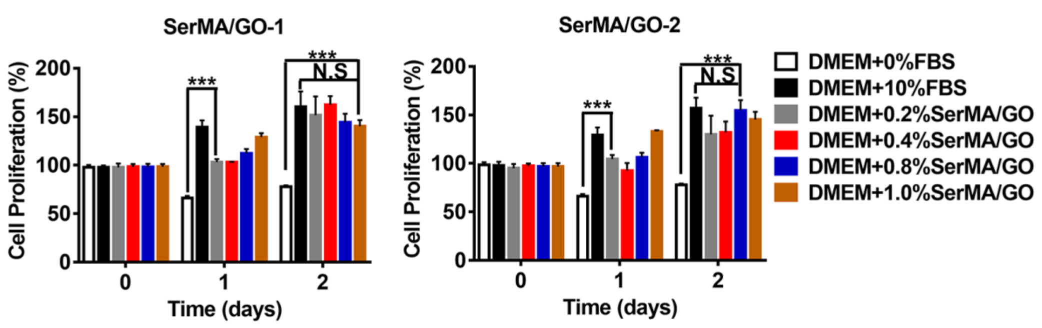

Figure S2. The cell proliferation of BMSCs co-cultured with SerMA/GO-1(Left) and SerMA/GO-2 (Right) after 24 and 48 hours. ***P<0.001, N.S: not significant. Three samples per group per condition.

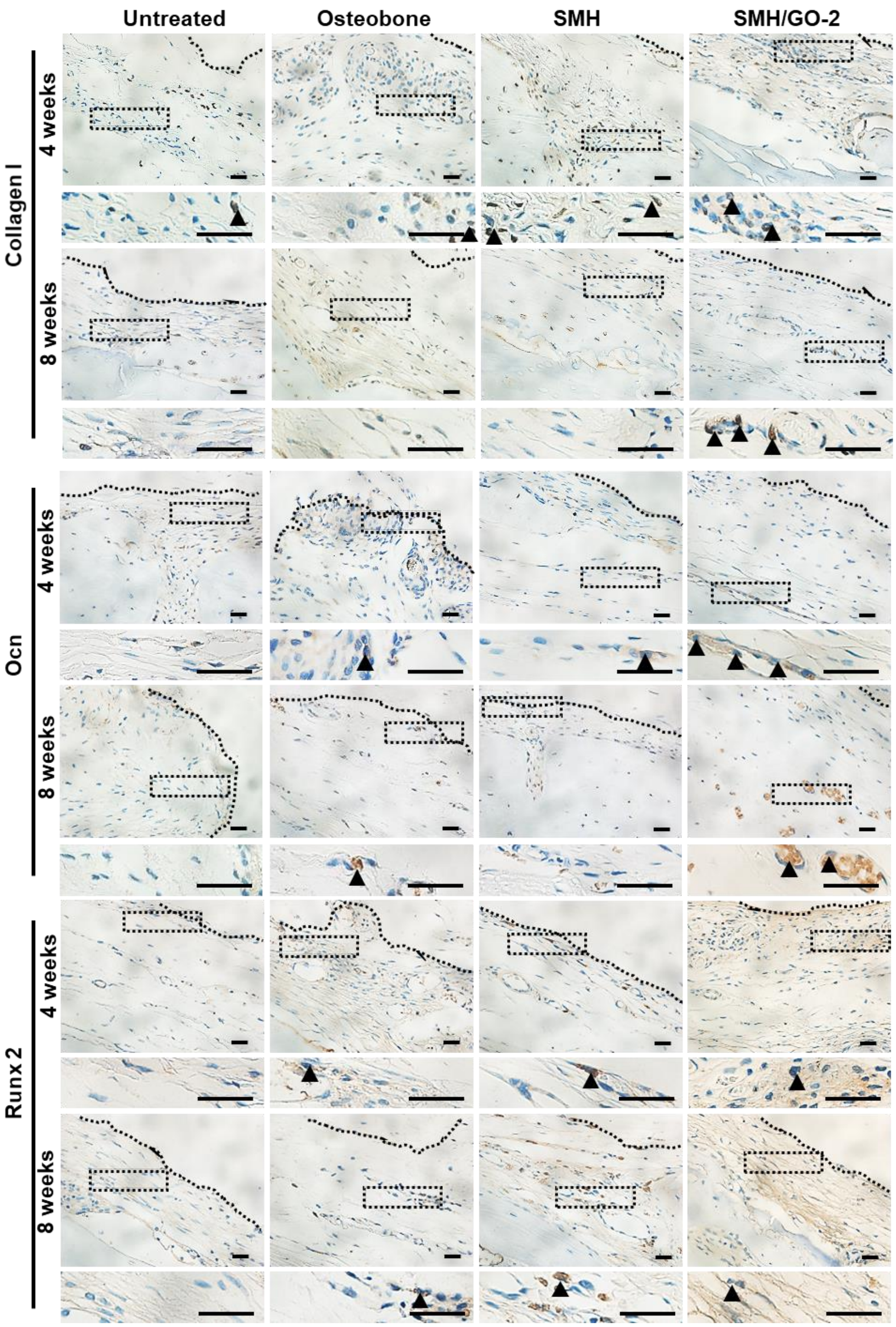

Figure S3. The immunohistological staining of Collagen I, Ocn and Runx 2 in wound sites treated by Osteobone, SMH and SMH/GO-2 4 and 8weeks after treatments. The black dotted boxes in the upper panels were enlarged in the lower panels. The black triangle indicates positive cell.

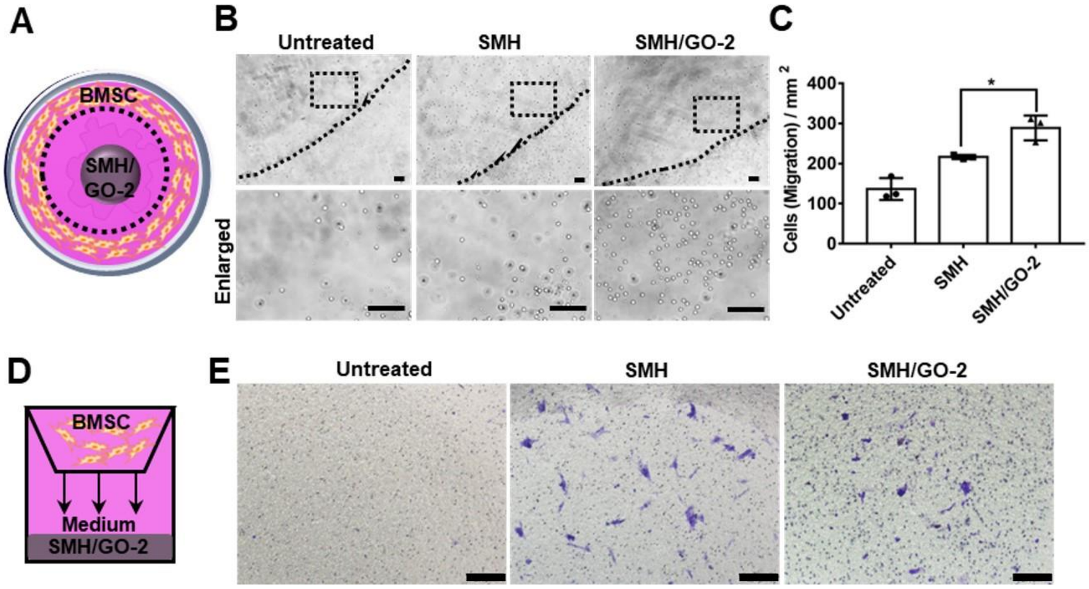

Figure S4. SMH/GO hydrogels promote BMSCs directional migration *in vitro*. (A) Schematics of directed migration assay where BMSCs seeded in the peripheral zone of the dish were co-cultured with SMH/GO-2 or SMH hydrogels that were placed at the center of the dish. (B) Directed migration assay of BMSCs co-cultured with SMH/GO-2 hydrogel or SMH hydrogel. The black dotted boxes in the upper panels were enlarged in the lower panels. The black dotted line indicated boundary of wound closure. Scale bars, 100 μm. (C) Quantification of migrated cells number in (B). (D) Schematics of transwell assay of BMSCs co-cultured with SMH/GO-2 or SMH hydrogels. Transwell assay of BMSCs was detected using 24-well Boyden chambers. BMSCs were seeded on the upper chamber of with serum-free IMDM medium, and IMDM medium containing SMH/GO hydrogel was added in the lower chamber. (E) Transwell assay of BMSCs co-cultured with SMH/GO-2 or SMH hydrogel. Migrated BMSCs were stained with crystal violet. *P<0.05. n=3 per group. Three random fields per groups were quantified. Scale bars, 100 μm.

Table S1. The primer sequences of Runx 2, Ocn, Col 1, GAPDH.

Gene	Species	Primers	
Runx 2	Rattus norvegicus	Forward	5’-TACCAGCCACCGAGACCAA-3’
		Reverse	5’-AGAGGCTGTTTGACGCCATAG-3’
Ocn	Rattus norvegicus	Forward	5’-GAGGACCCTCTCTCTGCTCA -3’
		Reverse	5’- GGTAGCGCCGGAGTCTATTC -3’
Col 1	Rattus norvegicus	Forward	5’-GGAGAGAGCATGACCGATGG -3’
		Reverse	5’-GGGACTTCTTGAGGTTGCCA -3’
GAPDH	Rattus norvegicus	Forward	5’-ATGACTCTACCCACGGCAAG -3’
		Reverse	5’-TACTCAGCACCAGCATCACC -3’

Table S2 Specific gene FPKM of BMSCs co-cultured with SMH/GO or Untreated in MAPK signaling pathway

MAPK signaling pathway			
Number	Gene Name	Untreated (FPKM)	SMH/GO(FPKM)
1	Il1b	1.623052	1401.488
2	Cd14	38.24301	514.6499
3	Met	5.165546	32.29564
4	Rac2	33.33944	134.1875
5	Pla2g4a	10.40573	40.57306
6	Il1a	3.661276	526.4951
7	Ereg	0.362251	13.45537
8	Rps6ka5	2.577283	11.7867
9	Nfkb2	16.27138	50.78408
10	Igf1	17.90147	18.347
11	Ngf	21.08205	66.50096
12	Flnc	41.40038	17.77473
13	Map3K8	3.158313	11.2547
14	Relb	8.087848	30.46607
15	Dusp2	1.175138	11.91962
16	Tgfbr1	26.76477	10.97179
17	Rapgef2	4.943177	13.93539
18	Erbb3	2.848232	0.523095
19	Stmn1	59.17106	23.44199
20	Angpt1	6.82256	27.52228
21	Mknk2	56.97755	28.70072
22	Mras	14.9577	28.22213
23	Mapkapk2	61.2313	98.86482
24	Cacna1h	0.546154	1.872956
25	Map2k1	103.5989	155.1018
26	Egfr	5.861101	13.33473
27	Vegfa	109.2931	218.2653
28	Rasgrp3	19.22667	9.852694
29	Pak1	55.90118	81.44923
30	Cdc25b	12.11904	5.970186
31	Mknk1	34.35821	59.34401
32	Dusp16	3.953068	8.667111
33	Rela	24.88723	38.2246
34	Mef2c	3.581978	1.783404
35	Rras2	43.02837	28.17038
36	Vegfb	20.16496	11.37763
37	Cacnb3	14.07856	23.23441
38	Tgfbr2	82.42284	60.45992

39	Rps6ka1	5.174784	9.937583
40	Vegfc	13.90169	21.86858
41	Map4k2	3.729266	6.347533
42	Hgf	4.438248	7.544394
43	Ppp3ca	20.22594	29.40477
44	Pdgfc	15.39575	9.111755
45	Pdgfb	2.032077	0.493755
46	Tnfrsf1a	66.23854	88.22742
47	Il1rap	4.642188	7.812615
48	Dusp1	134.577	87.26142
49	Arrb1	13.55738	8.641113
50	Tp53	52.76163	74.01943
51	Traf2	12.6105	18.99356
52	Myd88	20.45117	29.61648
53	Rps6ka2	7.379798	4.659369
54	Rasgrp4	3.286303	1.244377
55	Kitlg	35.9552	26.27229
56	Tek	0.203608	0.743617
57	Tgfb1	87.00561	135.1933
58	Map4k4	60.78542	80.40064
59	Rras	152.9111	212.695
60	Rasgrp1	0.683354	4.381518
61	Rps6ka3	9.805472	7.220897
62	Map3k5	4.476667	2.818381
63	Rap1b	259.2907	340.0331
64	Prkca	5.558527	3.680107
65	Map3k6	3.680808	5.511203
66	Mapkapk3	16.61232	24.8215
67	Lamtor3	39.26541	28.9596
68	Il1r1	27.38465	59.78841
69	Ddit3	56.42943	38.85496
70	Flnb	17.59144	13.54834
71	Gna12	58.9272	71.67332
72	Gadd45b	37.01894	55.87612
73	Map3k4	2.99085	1.919564
74	Jun	37.94804	27.64929
75	Tgfb3	340.8784	119.8837
76	Mapk1	67.93591	54.66568
77	Traf6	3.578344	5.509127
78	Dusp5	16.60769	12.16619
79	Vegfd	4.527468	6.764722
80	Mapk14	29.09758	23.62747
81	Cacng4	0.462309	0.10805
82	Rasa1	27.28143	22.35787

83	Dusp7	14.32514	18.5212
84	LOC108348108	3.059511	1.524943
85	Rasgrp2	0.793867	0.22073
86	Fgf7	322.7311	407.612
87	Map4k1	2.645767	1.557025
88	Casp3	15.3033	19.67364
89	Nr4a1	29.59364	22.0345
90	Ptpn7	6.359351	4.595449
91	Epha2	9.561579	12.64939
92	Efna5	10.15423	15.582
93	Ntrk1	0.258569	0.684931
94	Araf	29.28109	35.48454
95	Flt1	0.077195	0.247406
96	Dusp3	24.87252	21.11105
97	Cdc42	284.8574	329.0382
98	Irak1	39.53599	33.66571
99	Dusp4	2.448055	1.618515
100	Cacng7	0.037575	0.319936
101	Pdgfrb	112.6887	98.66304
102	Dusp6	20.96846	25.63562
103	Atf4	139.4125	159.1158
104	Map3k1	0.647811	0.314476
105	Kras	18.25147	14.36808
106	Rps6ka4	27.96639	23.04429
107	Ppm1a	26.42435	31.38105
108	Cacna2d1	8.107261	10.21977
109	Map3k20	24.46194	20.16484
110	Pla2g4d	0.146171	0.486348
111	LOC103694380	0.143452	0.83006
112	Hspb1	353.8885	203.5138
113	Ppp3r1	38.17381	43.49968
114	Angpt4	22.73993	18.84271
115	Mapk3	76.51053	89.31088
116	Rac1	123.4613	138.122

FPKM: Fragments Per Kilobase of transcript per Million fragments mapped

Table S3 Specific gene FPKM of BMSCs co-cultured with SMH/GO or Untreated in TNF signaling pathway

TNF signaling pathway			
Number	Gene Name	Untreated (FPKM)	SMH/GO(FPKM)
1	Il1b	1.623052	1401.488
2	Ccl12	21.16123	553.2132
3	Mmp9	11.70843	154.1042
4	Ptgs2	56.17974	460.6985
5	Tnfaip3	2.801209	26.95747
6	Creb3l1	19.1176	89.07108
7	Birc3	2.746906	19.0014
8	Nod2	0.831215	8.9331
9	Tnfrsf1b	10.2201	54.22913
10	Cflar	14.91256	56.11503
11	Rps6ka5	2.577283	11.7867
12	Ccl2	775.2646	3705.978
13	Nfkb1	18.99323	53.76932
14	Icam1	52.80183	128.9413
15	Cxcl2	15.19772	655.1574
16	Cxcl3	107.857	1726.01
17	Csf2	1.05409	32.93001
18	Map3k8	3.158313	11.2547
19	Mmp14	255.0856	547.3885
20	Traf1	1.485267	7.088363
21	Socs3	31.08256	192.4548
22	Il6	41.1276	381.4086
23	Map2k1	103.5989	155.1018
24	Lif	8.625385	59.89222
25	Mmp3	82.69355	659.1337
26	Jag1	5.827515	13.60523
27	Cxcl1	363.9489	1743.355
28	Ccl20	32.31175	838.0274
29	Rela	24.88723	38.2246
30	Birc2	27.76928	18.87565
31	Creb5	0.530428	1.729823
32	Pik3cb	5.225934	8.911469
33	Traf3	10.5922	14.71402
34	Tnfrsf1a	66.23854	88.22742
35	Bcl3	6.345241	26.49079
36	Traf2	12.6105	18.99356
37	Ifi47	7.53558	4.051224
38	Map3k5	4.476667	2.818381

39	Casp8	6.68462	10.28108
40	Junb	90.5224	206.4717
41	Cxcl10	1.852138	0.364506
42	Cebpb	99.43952	224.4731
43	Il15	7.793793	15.20702
44	Jun	37.94804	27.64929
45	Ccl5	11.33922	41.83816
46	Mapk1	67.93591	54.66568
47	Il18r1	0.331079	1.022176
48	Pik3r3	7.46865	4.703522
49	Mapk14	29.09758	23.62747
50	Creb3l2	22.18448	17.78673
51	Pik3ca	7.449071	9.307282
52	Casp3	15.3033	19.67364
53	Ripk1	12.22232	14.92071
54	Atf4	139.4125	159.1158
55	Rps6ka4	27.96639	23.04429
56	Atf6b	41.4469	49.12532
57	LOC103694380	0.143452	0.83006
58	Mapk3	76.51053	89.31088
59	Irf1	7.313214	9.409684

Table S4 Specific gene FPKM of BMSCs co-cultured with SMH/GO or Untreated in chemokine signaling pathway

Chemokine signaling pathway			
Number	Gene Name	Untreated (FPKM)	SMH/GO(FPKM)
1	Ccl3	8.332658	657.1035
2	Ccl4	7.360073	658.7879
3	Ccl12	21.16123	553.2132
4	Ccl9	4.82618	157.4049
5	Nfkbia	18.3863	157.3239
6	Cxcl16	24.9772	123.0666
7	Jak2	16.82202	66.58971
8	Rac2	33.33944	134.1875
9	Ccl2	775.2646	3705.978
10	Gng12	117.78	290.8318
11	Nfkb1	18.99323	53.76932
12	Cxcl2	153.345	192.8504
13	Ptk2b	14.12786	35.08393
14	Ccl7	621.3104	2311.426
15	Cxcl3	107.857	1726.01
16	Gngt2	87.00516	27.25305
17	Stat4	14.98958	6.81881
18	Fgr	10.53057	24.45835
19	Gng8	17.96435	50.58306
20	Hck	33.1809	70.7582
21	Ccl6	6.202822	244.713
22	Ncf1	42.86032	86.82888
23	Nfkbib	9.155127	25.92058
24	Pik3cg	5.106987	12.65649
25	Pik3r5	8.159415	18.09959
26	Plcb2	6.77266	2.800457
27	Map2k1	103.5989	155.1018
28	Adcy7	21.79331	36.89955
29	Lyn	26.06949	44.98457
30	Pak1	55.90118	81.44923
31	Prex1	10.86415	19.77547
32	Cxcl1	363.9489	1743.355
33	Src	23.78335	35.33737
34	Pf4	1528.487	2652.949
35	Ccl20	32.31175	838.0274
36	Rela	24.88723	38.2246
37	Prkcd	23.52993	34.90354
38	Pxn	23.72752	16.26606

39	Pik3cb	12.85879	11.15998
40	Stat3	69.22348	90.36574
41	Cxcr4	19.08462	29.37529
42	Arrb1	13.55738	8.641113
43	Shc1	82.813	106.674
44	Tiam1	4.697278	7.27088
45	Cxcl6	157.3477	750.4836
46	Adcy8	1.350699	3.516613
47	Adcy2	1.674548	3.534729
48	Rap1b	259.2907	340.0331
49	Rock2	49.99281	38.40963
50	Ccr5	8.691501	4.481508
51	Cxcl10	1.852138	0.364506
52	Vav2	8.972782	6.65675
53	Gnb5	15.23428	21.96619
54	Gng10	143.397	173.7987
55	Plcb4	10.56106	7.889541
56	Stat2	13.73429	17.58303
57	Ccl5	11.33922	41.83816
58	Mapk1	67.93591	54.66568
59	Grk6	13.101	10.26047
60	Pik3r3	7.46865	4.703522
61	Ppbp	0.277834	1.602305
62	Adcy4	14.54621	10.86936
63	Pik3r6	0.32567	0.030944
64	Rasgrp2	0.793867	0.22073
65	Adcy9	2.388793	1.468331
66	Gng2	8.394958	11.15021
67	Pik3ca	7.449071	9.307282
68	Elmo1	4.113742	2.516399
69	Cdc42	284.8574	329.0382
70	Gsk3b	28.82657	34.82599
71	Kras	18.25147	14.36808
72	LOC108349548	95.29955	81.68275
73	Cxcl11	1.191536	2.222097
74	Cxcl14	8.859395	11.53209
75	Cx3cr1	0.206636	0
76	Gnai1	26.33932	21.59448
77	Gng4	0.094157	0.331537
78	Mapk3	76.51053	89.31088
79	Rac1	123.4613	138.122
